# Walter Everaerd (1937–2019)

**DOI:** 10.1007/s10508-020-01659-1

**Published:** 2020-02-18

**Authors:** Ellen Laan, Erick Janssen

**Affiliations:** 1 Department of Sexology and Psychosomatic Gynaecology, Amsterdam University Medical Center, University of Amsterdam, Meibergdreef 9, 1105 AZ Amsterdam, The Netherlands; 2 Department of Neurosciences, Institute for Family and Sexuality Studies, University of Leuven, Louvain, Belgium

Walter Everaerd, former President of the International Academy of Sex Research, World Association for Sexual Health (WAS) gold medalist, and internationally acclaimed sex researcher, died on October 4, 2019, at the age of 82. Walter had been suffering from bladder cancer for several years. He is survived by his wife Bets, his three children, and eight grandchildren.

Walter was a pioneer in experimental sexology in the Netherlands. At the age of 21, after two years of military service, Walter began his studies at Utrecht University, where he specialized in the new and promising fields of experimental clinical psychology and psychotherapy. In an academic environment that was at the time mainly oriented toward psychoanalysis and introspection as scientific methods, Walter approached clinical problems as an experimental psychologist and behavioral therapist. He became a full professor in Psychology at the age of 37 at Utrecht University, and he moved to the University of Amsterdam 12 years later. Over the years, with his students and collaborators, he received numerous grants in fields as diverse as symptom perception, interoception, vaginismus, sexual arousal, stress and health, cancer, asthma, and traumatic memory. Walter’s contributions to the field of sexology, both theoretically and experimentally, have significantly changed and advanced how we define sexuality and treat sexual problems. Influenced by Walter’s work, the field of sexuality has now moved away from drive and libido as the main explanatory constructs for sexual response and sexual expression toward information processing models of sexual response. Walter was the first scholar to propose that sexual arousal should be considered an emotion. Also, current views and knowledge of sexual desire and sexual arousal problems, as reflected by the DSM-5, have, in more direct and indirect ways, been informed and inspired by Walter’s theoretical and experimental work.

Walter was a modest and kind man who took his task as an academic mentor as seriously as his scientific endeavors. He mentored almost 60 Ph.D. students, many of whom became full professors. It is probably no exaggeration to say that he raised several generations of Dutch sexologists. His former Ph.D. students include Stephanie Both, Peggy Cohen-Kettenis, Joost Dekker, Jos Frenken, Luk Gijs, Erick Janssen, Bram Kuiper, Ellen Laan, Anne Oldenhave, Theo Sandfort, Mark Spiering, Arno Toorians, Adriaan Tuiten, Daan van Beek, Janneke van der Velde, and Jacques van Lankveld. Walter was honorary member, scientific program chair, and host and president of several national and international psychological and sexological societies, including the International Academy of Sex Research. He also served as department chair and as a board member and chair of various university bodies and national organizations, such as the Dutch Association for Gay and Lesbian Studies and the Dutch Health Council. He was the recipient of many awards, including the Heymans Award of the Dutch Psychological Association, the Sexologist of the Year Award of the Polish Academy of Sexology, the van Emde Boas–van Ussel Award of the Dutch Society of Sexology, and the WAS Gold Medal.

We are grateful for Walter’s many contributions, and we will miss him dearly.

